# Multi-night cortico-basal recordings reveal mechanisms of NREM slow wave suppression and spontaneous awakenings at high-temporal resolution in Parkinson’s disease

**DOI:** 10.21203/rs.3.rs-3484527/v1

**Published:** 2023-11-07

**Authors:** Md Fahim Anjum, Clay Smyth, Derk-Jan Dijk, Philip Starr, Timothy Denison, Simon Little

**Affiliations:** 1 Movement Disorders and Neuromodulation Centre, University California San Francisco, CA, USA; 2 Surrey Sleep Research Centre, University of Surrey, Guildford, United Kingdom.; 3 UK Dementia Research Institute, Care Research and Technology Centre at Imperial College, London and the University of Surrey, Guildford, United Kingdom.; 4 MRC Brain Network Dynamics Unit, University of Oxford, United Kingdom

## Abstract

**Background:**

Sleep disturbance is a prevalent and highly disabling comorbidity in individuals with Parkinson’s disease (PD) that leads to worsening of daytime symptoms, reduced quality of life and accelerated disease progression.

**Objectives:**

We aimed to record naturalistic overnight cortico-basal neural activity in people with PD, in order to determine the neurophysiology of spontaneous awakenings and slow wave suppression in non-rapid eye movement (NREM) sleep, towards the development of novel sleep-targeted neurostimulation therapies.

**Methods:**

Multi-night (n=58) intracranial recordings were performed at-home, from chronic electrocorticography and subcortical electrodes, with sensing-enabled Deep Brain Stimulation (DBS), paired with portable polysomnography. Four participants with PD and one participant with cervical dystonia were evaluated to determine the neural structures, signals and functional connectivity modulated during NREM sleep and prior to spontaneous awakenings. Intracranial recordings were performed both ON and OFF DBS to evaluate the impact of stimulation. Sleep staging was then classified with machine-learning models using intracranial cortico-basal signals on classical (30 s) and rapid (5 s) timescales.

**Results:**

We demonstrate an increase in cortico-basal slow wave delta (1–4 Hz) activity and a decrease in beta (13–31 Hz) activity during NREM (N2 and N3) versus wakefulness in PD. Cortical-basal ganglia coherence was also found to be higher in the delta range and lower in the beta range during NREM. DBS stimulation resulted in a further elevation in cortical delta and a decrease in alpha (8–13 Hz) and low beta (13–15 Hz) power compared to the OFF stimulation state. Within NREM sleep, we observed a strong inverse interaction between subcortical beta and cortical slow wave activity and found that subcortical beta increases prior to spontaneous awakenings at high-temporal resolution (5s). Our machine-learning models trained on intracranial cortical or subcortical power features achieved high accuracy in both traditional (30s) and rapid (5s) time windows for NREM vs. wakefulness classification (30s: 92.6±1.7%; 5s: 88.3±2.1%).

**Conclusions:**

Chronic, multi-night recordings in PD reveal increased cortico-basal slow wave, decreased beta activity, and changes in functional connectivity in NREM vs wakefulness, effects that are enhanced in the presence of DBS. Within NREM, subcortical beta and cortical delta are strongly inversely correlated and subcortical beta power increases prior to spontaneous awakenings. Our findings elucidate the network-level neurophysiology of sleep dysfunction in PD and the mechanistic impact of conventional DBS. Additionally, through accurate machine-learning classification of spontaneous awakenings, this study also provides a foundation for future personalized adaptive DBS therapies for sleep dysfunction in PD.

## Introduction

Sleep disruption is one of the most prevalent non-motor symptoms of Parkinson’s disease (PD) with up to 90% of PD patients experiencing sleep dysfunction^1^ and 60% having multiple sleep disturbance symptoms^1,2^. Sleep dysfunction in PD has a negative impact on daytime mood, cognition, fatigue, and other co-morbidities^3–7^, with non-motor and sleep-related symptoms being a greater determinant of quality of life than classical motor symptoms^8–10^. Changes in sleep patterns often predate classical neurological symptoms in PD and overnight slow wave dysfunction correlates with rates of disease progression and severity^11,12^. Therefore, understanding the neurophysiology of sleep disturbances in PD may potentially result in new principled therapies directed towards better sleep quality, mitigation of daytime symptoms, improved patients’ quality of life and the development of therapeutic targets for disease progression modification.

Sleep architecture in humans is broadly defined by physiologically distinct stages of rapid eye movement (REM) and non-REM (NREM) sleep. NREM sleep is further characterized by rhythmic low-frequency electroencephalography (EEG) activity in the delta (0–4 Hz) and theta (4–8 Hz) ranges, increased parasympathetic activity, and limited dreaming. There are currently three formally defined sub-stages of NREM: N1 (light sleep), N2 (appearance of K complexes and sleep spindles) and N3 (characterized by slow delta waves)^13^. Sleep dysfunction in PD manifests as parasomnias, fragmented sleep and disrupted sleep patterns, including notable reductions in both REM and NREM sleep^10^. In particular, reductions in NREM slow wave activity in the delta range (< 4Hz) are associated with worsening of daytime motor symptoms and accelerated disease progression in PD^11,14,15^.

During wakefulness, beta oscillations (13–31 Hz) are the hallmark oscillatory signature of PD and correlate with daytime motor symptoms^16^. Recent studies with non-human primates (NHPs) during sleep have shown that subcortical beta activity is also associated with a decrease in cortical delta activity and suggested a role for subcortical beta in spontaneous awakenings in PD^17^. Indeed, the presence of subcortical beta oscillations has been detected during sleep in PD patients^18–23^. However, to date, human studies have not yet investigated mechanistic interactions between subcortical beta and cortical sleep physiology (inc. slow waves) nor spontaneous awakenings within PD subjects. Previous studies have been limited to single-night, across-subject analyses that have not yet determined if subcortical beta is a reliable biomarker of awakening within subjects, or in the presence of deep brain stimulation (DBS).

Understanding the real-world contribution of the cortico-basal ganglia circuit to sleep dysfunction in PD and its interaction with DBS has been limited by an inability to chronically record the intracranial activities overnight, at high resolution. This challenge has been solved by the advent of a new generation of sensing-enabled DBS devices that can stream neural data remotely from participants’ own homes^24^. A better understanding of cortico-basal activities during sleep has the potential to reveal underlying mechanisms of sleep dysfunction in PD and could contribute to improved sleep therapies including sleep-targeted adaptive deep brain stimulation (aDBS) for neurological disorders^25^.

In this study, we recruited four participants diagnosed with PD and one comparison participant with cervical dystonia, all with chronically implanted intracranial electrodes capable of sensing sensorimotor cortical and basal ganglia (subthalamic nucleus: STN / globus pallidus: GPi) field potentials (FPs). We conducted large data collection, within-subject, multi-night, at-home, intracranial cortical and subcortical recordings paired with portable polysomnography over multiple nights (n= 58) in the presence and absence of DBS stimulation. We demonstrate significant negative interactions between subcortical beta oscillations and cortical slow wave activity in the delta band during N2/N3 NREM, an effect modulated by DBS, and also show that subcortical beta significantly increases prior to spontaneous awakenings at high-temporal resolution (5s time window). Finally, we demonstrate successful classification of N2/N3 NREM vs wakefulness in both classical (30s) and sub-classical (5s) time windows using constrained machine-learning approaches using bandpower features from intracranial neural recordings - towards the development of sleep-specific adaptive DBS therapies for neurological disorders.

## Results

**Figure 1.**
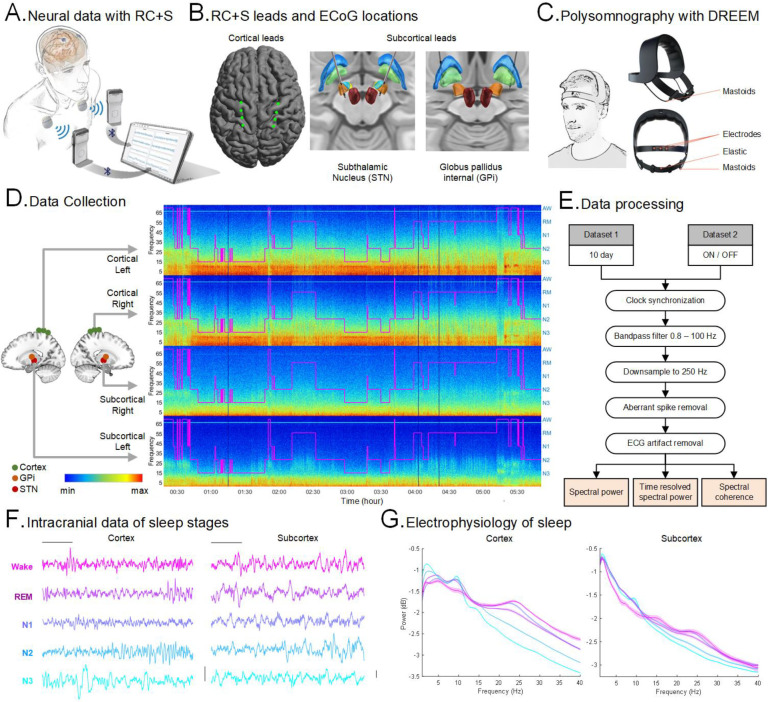
Methodology, data collection and analysis procedures: **(A)** Schematic of the RC+S system setup for recording intracranial cortical Field Potentials (FP) in participants (Adapted from Gilron et al. 2021^24^). **(B)** Illustrations of the placement of RC+S sensing depth electrodes in subcortex for both STN and GPi (*right*) and cortical ECoG locations (*left*). Example data from PD2 and PD3 participants. **(C)** Schematic of the Dreem2, portable headband for recording in-home polysomnography overnight (adapted from Debellemaniere et al. 2018^39^). **(D)** Illustration of a single night of sleep in a PD participant (DBS ON) with hypnogram (purple) showing sleep stages (AW: awake; RM: REM; [N1, N2, N3]: NREM) and cortical (top 2 panels) and subcortical (bottom 2 panels) spectrogram panels from both hemispheres showing multi-frequency changes across sleep stages where the x-axis is time in hours and y-axis is frequency (Hz). FP was recorded bilaterally from cortical and subcortical regions. **(E)** Flowchart of data analysis and preprocessing procedures for multi-night sleep dataset (n=5) and ON/OFF dataset (n=4). **(F)** Representative traces of the RC+S FP time series in all sleep stages from cortex (*left column*) and subcortex (*right column*; Subthalamic Nucleus). Columns share scale bars and rows share color legends (Wake, REM, N1, N2 and N3). Data from one PD participant with ON stimulation from the left hemisphere. **(G)** Comparisons of spectral powers of intracranial FPs among sleep stages in cortex (*left*) and subcortex (*right*) for a single participant, DBS ON. Shaded error bars indicate standard error. Shares color legend with panel F.

Four participants (Table 1) with PD (x2 with bilateral STN + sensorimotor cortical ECoG and x2 with bilateral GPi electrodes + sensorimotor cortical ECoG) and one participant with cervical dystonia (bilateral GPi electrodes + sensorimotor cortical ECoG), successfully initiated recordings from intracranial cortico-basal and external portable polysomnography (Dreem2^26,27^) over a total of 58 nights (54 ON and 4 OFF stimulation nights), remotely in their own homes. Intracranial and extracranial recordings were synchronized and artifacts were removed (Fig. 1; Supplementary Fig. 3), resulting in interpretable cortical and subcortical recordings, even in the presence of DBS. A total 415 hours of sleep were recorded across all participants (Supplementary Table 1; Supplementary Fig. 1). PD participants slept on average 7.25±0.18 hours per night during the multi-night ON stimulation recording phase (n=45; total duration in minutes per night: N1 = 34.26±1.34; N2 = 164.72±7.92; N3 = 90.06±10.53; REM = 97.64±6.54; Wake after sleep = 48.18±4.71). In a separate two-night, consecutive ON versus OFF DBS comparison, all four PD participants showed an increase in time in deep N3 NREM and REM in the ON stimulation compared to the OFF stimulation nights (Supplementary Table 1; Supplementary Fig. 2). Power spectral density plots from intracranial electrodes (Fig. 1G and Supplementary Fig. 4) demonstrated expected classical changes in canonical frequency bands in NREM and REM sleep stages, supporting appropriate dissociation of different sleep stages using portable PSG device sleep staging.

### Spectral power changes in NREM

**Figure 2.**
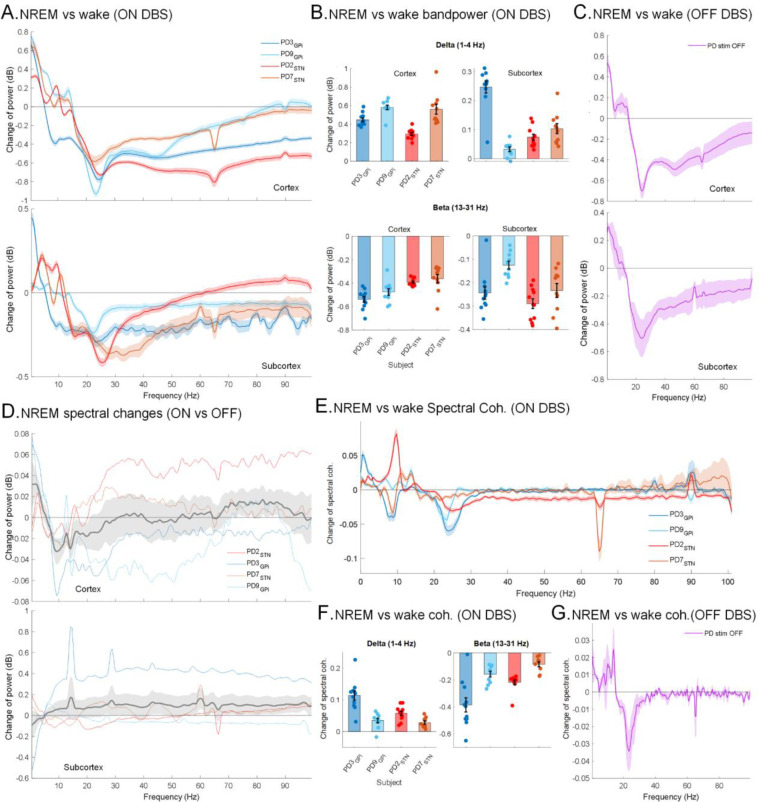
Dynamic changes in power spectra and functional connectivity between cortical and subcortical regions during N2/N3 NREM sleep: **(A)** Power Spectrum changes (mean ± SEM) during N2/N3 NREM sleep with wake stage as baseline in low frequency range (1–50 Hz) for all PD participants (n=4) during ON stimulation in cortical (*top*) and subcortical (*bottom*) areas. y-axis shows the difference in power spectra between N2/N3 NREM and wake stage in decibels (dB). Thick lines show mean and shaded areas show standard errors (SEM). **(B)** Power in delta (1–4 Hz) increases while beta (13–31 Hz) band power decreases during N2/N3 NREM sleep compared to wake during ON stimulation in cortical (*top*) and subcortical (*bottom*) areas. Each bar shows the difference in spectral power for one participant averaged across multiple nights and each data point shows the average difference in spectral power across one night with data pooled from both hemispheres. **(C)** During OFF stimulation conditions, delta power increases while beta power decreases in N2/N3 NREM compared to the wake stage in PD participants (n=4) in cortical (*top*) and subcortical (*bottom*) areas. Thick lines show means and shaded areas show standard errors. **(D)** Difference in cortical spectral power between ON and OFF stimulation conditions in 4 participants with PD in N2/N3 NREM sleep stages (*top*), showing increased delta (1–4 Hz) and decreased alpha and sigma activities (8–15 Hz) while ON stimulation. Each colored line shows spectral change for one participant, thick line shows average across the participants with shaded area as SEM. The spectral power in subcortical regions didn’t show any statistically significant difference (*bottom*). The x-axis is frequency (Hz) and the y-axis is difference in power (ON-OFF). **(E)** Changes in cortical-subcortical spectral coherence (mean ± SEM) during N2/N3 NREM sleep with wake stage as baseline for all participants (n=5) during ON stimulation. y-axis shows the difference in spectral coherence between N2/N3 NREM and wake stage. Horizontal back line at 0 represents wake stage baseline. **(F)** Total difference in spectral coherence in delta (1–4 Hz, *left*) and beta (13–31 Hz, *right*) during N2/N3 NREM sleep compared to wake during ON stimulation. Each bar shows difference in spectral coherence for one participant averaged across multiple nights and each point shows average difference in spectral coherence across one night with data pooled from both hemispheres. **(G)** During OFF stimulation conditions, delta coherence increases while beta coherence decreases in N2/N3 NREM compared to the wake stage in PD participants (n=4). Data from both hemispheres were pooled for all panels.

We investigated overnight spectral changes in intracranial FP activities, specifically investigating the hypothesis that there is a negative interaction between cortico-basal delta and beta during N2/N3 NREM^28^. Power spectrum analyses and Linear Mixed Effect (LME) models for average overnight band powers with a fixed effect for sleep stage (N2/N3 NREM vs Wake; accounting for multiple nights within participants) and a random effect for participants (n=5) showed an increase in average delta power (1 – 4 Hz; cortex: ***β*** = 0.42, 95%CI= [0.38, 0.47], p-value = 3.7e-33; subcortex: ***β*** = 0.1, 95%CI= [0.07, 0.13], p-value = 3.3e-12; n=105; CI=confidence interval) and decrease in beta (13 – 31 Hz; cortex: ***β*** = −0.4, 95%CI= [−0.44, −0.37], p-value = 1.5e-41; subcortex: ***β*** = −0.2, 95%CI= [−0.22, −0.17], p-value = 1.4e-25) power both in cortical and subcortical regions in N2/N3 NREM sleep compared to wakefulness (Fig. 2A–B; multi-night ON stimulation). These spectral changes in N2/N3 NREM compared to wake were also observed during the single night of OFF DBS sleep recordings in both cortical and subcortical regions of all four PD participants (Fig. 2C).

A direct comparison of PD (n=4) vs Dystonia (n=1) revealed that subcortical beta power was lower in the dystonia than all four of the PD participants during N2/N3 NREM sleep (LME model for PD vs Dystonia fixed effect: ***β*** = 0.18; 95%CI= [0.09, 0.27]; p-value = 0.0001). Further, band power changes between N2/N3 NREM sleep and wakefulness conditions in the dystonia participant were smaller compared to the PD participants. Indeed, LME models demonstrated statistically significant fixed effects of disease state (PD vs Dystonia) on the changes of band power between N2/N3 NREM and wake stage in cortex (delta: ***β*** = 0.24, 95%CI= [0.14, 0.35], p-value = 1.3e-5; beta: ***β*** = −0.21; p-value = 3.4e-7) and subcortex (delta: ***β*** = 0.08, 95%CI= [0.013, 0.14], p-value = 0.02; beta: ***β*** = −0.13, 95%CI= [−0.2, −0.07], p-value = 0.0001), supporting more pronounced N2/N3 NREM vs. wake changes in PD vs Dystonia.

We also investigated how these FP activities alter with DBS during N2/N3 NREM and compared power spectrums between ON and OFF stimulation conditions in our PD cohort (n=4; Table 1). Spectral power comparisons revealed a relative further increase in delta and further decrease in alpha and sigma activities in cortical FP during N2/N3 NREM sleep in the ON versus OFF DBS conditions (Fig. 2D). Indeed, LME models with participants as random effects revealed that stimulation (ON DBS) resulted in a further increased cortical delta (1–4 Hz; ***β*** = 0.026, 95%CI= [0.003, 0.05], p-value = 0.03) and decreased cortical alpha (8–13 Hz; ***β*** = −0.0297, 95%CI= [−0.05, −0.003], p-value = 0.03) as well as sigma (13–15 Hz; ***β*** = −0.026, 95%CI= [−0.042, −0.01], p-value = 0.006) in N2/N3 NREM for the ON versus OFF condition. No significant changes in subcortical FP during N2/N3 NREM were observed in ON vs OFF power spectrum comparisons, however, changes in subcortical baseline power levels in the ON versus OFF DBS state may have obscured any underlying changes. Overall, these data reveal that DBS results in relatively higher cortical delta activity and reduced alpha and low beta activities in N2/N3 NREM sleep.

### Changes in functional connectivity in NREM

We next explored NREM-related changes in the functional connectivity between sensorimotor cortical and subcortical regions to investigate sleep-related changes in cortico-basal ganglia circuitry in PD. For this, we compared the spectral coherence in cortical and subcortical FP activities between N2/N3 NREM sleep and wakefulness. In all participants, LME models investigating spectral coherence with a fixed effect of sleep stage (N2/N3 NREM vs Wake) revealed that the total difference in spectral coherence in delta increases (***β*** = 0.05, 95%CI = [0.04, 0.06], p-value = 5e-11; n=104) while in beta decreases (***β*** = −0.18; 95%CI = [−0.23, −0.14], p-value = 5.5e-13) during N2/N3 NREM sleep compared to wake, ON stimulation (Fig. 2E–F). An increase in delta coherence and a decrease in beta coherence during N2/N3 NREM were also observed in the PD participants during their single night recordings OFF stimulation (Fig. 2G). PD vs Dystonia comparison also showed that cortico-basal delta/beta coherence changes in N2/N3 NREM versus wakefulness were smaller in the dystonia participants compared to the PD participants (LME model with PD/Dystonia condition as a fixed effect; delta coherence: ***β*** = 0.05, 95%CI= [0.02, 0.08], p-value = 0.0008; beta coherence: ***β*** = −0.21, 95%CI= [−0.31, −0.11], p-value = 0.0001).

In our ON vs OFF DBS analysis (PD participants only; n=4; Table 1), we also noted a statistically significant further decrease in cortico-basal sigma (13 – 15 Hz) coherence during ON stimulation compared to OFF (LME model with ON/OFF condition as fixed and participants as random effects; ***β*** = −0.012, 95%CI= [−0.021, −0.003], p-value = 0.015) during N2/N3 NREM. Collectively, these data demonstrate that functional connectivity between cortical and subcortical structures is modulated during N2/N3 NREM sleep versus wakefulness. There is an increase in delta coherence and a decrease in beta coherence in PD during N2/N3 NREM in both ON and OFF stimulation conditions, effects that are enhanced in the DBS ON condition.

### Interaction between cortical delta and subcortical beta activity

**Figure 3.**
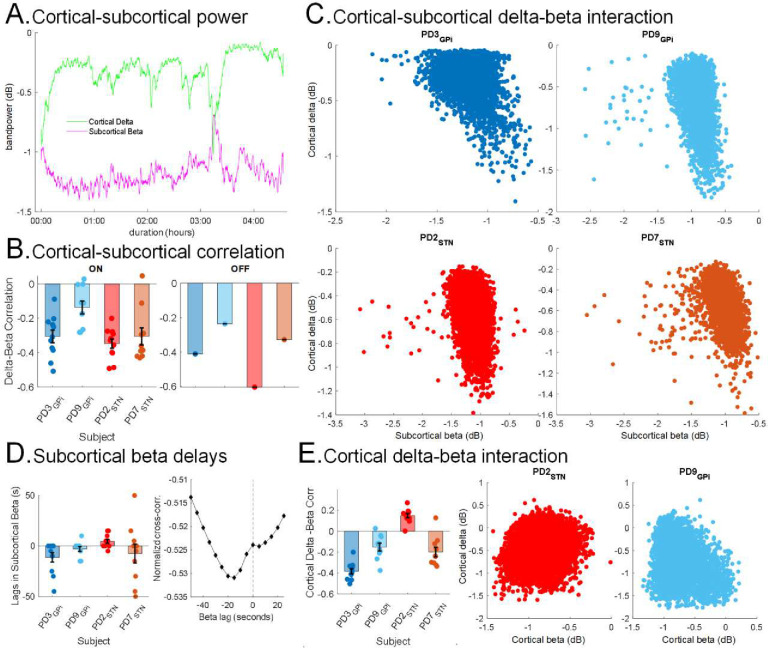
Inverse relationship between subcortical beta and cortical delta FP activities during N2/N3 NREM: **(A)** Example of subcortical beta (*purple*) and cortical delta (*green*) power during N2/N3 NREM in a single night from one PD participant (PD3) during ON stimulation depicting the inverse relationship in temporal domain. The delta and beta powers were smoothed with a 20-point gaussian kernel. **(B)** Average Spearman’s rho correlation between subcortical beta power and cortical delta power for all 4 PD participants across multiple nights in ON (*left*) and in OFF (*right*) stimulation during N2/N3 NREM. Each bar shows average correlation for one participant and each point shows correlation across one night with data pooled from both hemispheres. **(C)** Scatter plots depicting the correlation between subcortical FP beta (13–31 Hz) power and cortical FP delta (1–4 Hz) power during N2/N3 NREM sleep in 4 PD participants during ON stimulation; STN (*brown and red*), and GPi (*blue, light blue*). Each point represents data from one 5s N2/N3 NREM sleep epoch. Each plot is data from one night pooled from both hemispheres for one participant. **(D)** Normalized cross-correlation between subcortical beta power and cortical delta power showing the subcortical beta preceding cortical delta activities in PD participants during N2/N3 NREM with ON stimulation. The bar plot (*left*) shows lags in subcortical beta with cortical delta as reference. Each bar shows average lag for one participant and each point shows lag across one night with data pooled from both hemispheres. Example of cross-correlation showing the lag in subcortical beta as a function of time (*right*) in one night from PD2 during ON stimulation. The vertical dashed line shows zero-lag. **(E)** Interactions between cortical delta and cortical beta activities, examined as a control for cortical delta-subcortical beta during N2/N3 NREM. The bar plot (*left*) shows average Spearman’s rho correlation between cortical delta and beta power for all 4 PD participants across multiple nights, ON stimulation. Each bar shows average correlation for one participant and each point shows correlation across one night with data pooled from both hemispheres. The scatter plots (*middle and right*) show cortical delta and beta power in 4 PD participants during ON stimulation for two representative PD participants. Each point represents data from one 5s N2/N3 NREM sleep epoch.

Spectral power and functional connectivity analyses above revealed opposing changes in delta and beta FP activities in N2/N3 NREM sleep versus wakefulness. To further examine for a direct relationship between these two rhythms, we investigated the interactions between cortical delta and subcortical beta FP activities specifically *within* N2/N3 NREM on shorter, within-sleep stage, time scales (5s). Here, we observed an inverse relationship between cortical delta power and subcortical beta power during N2/N3 NREM sleep (Fig. 3A). To quantify this relationship, we first used a standard correlation analysis which revealed a negative correlation between subcortical beta and cortical delta FP power (5s epochs) in all PD participants during N2/N3 NREM in both ON and OFF stimulation conditions (Fig. 3B–C). LME modeling using band powers of N2/N3 NREM epochs from all participants (Cervical dystonia and PD participants; accounting for the dependency between left and right hemispheres and multiple nights within participants; n=232,064) showed an overall negative fixed effect of subcortical beta power on cortical delta power (***β*** = −0.24, 95%CI: [−0.28, −0.2], p-value = 3.9e-30) during N2/N3 NREM sleep, ON stimulation. Additionally, the LME model revealed a fixed effect of PD vs Dystonia state (***β*** = 0.06, 95%CI: [0.01, 0.11], p-value = 0.02), demonstrating that this effect was greater in the PD participants than our dystonia comparison participant. A negative fixed effect of subcortical beta power on cortical delta power was also obtained through an LME model in PD participants during N2/N3 NREM in the OFF stimulation condition (***β*** = −0.38, 95%CI: [−0.44, −0.32], p-value = 1.9e-32; n=17,518). These results demonstrate that there is an inverse relationship between subcortical beta and cortical delta FP power within N2/N3 NREM sleep in PD both during ON and OFF stimulation conditions and that this effect is significantly stronger than in our comparison dystonia participant.

Next, we utilized cross-correlation analyses to determine whether subcortical beta was leading or lagging cortical delta changes. We observed that the subcortical beta increase was leading the cortical delta decrease in 3 out of the 4 PD participants during N2/N3 NREM sleep (Fig. 3D; average lag over multiple nights ON DBS; PD2: 4.5s, n=11; PD3: −11.4s, n=11, PD7: −7.5s, n=10, PD9: −3s, n=10). Finally, as a control analysis to rule out a prosaic inverse relationship between cortico-basal circuit delta and beta, simply reflecting the depth of NREM sleep, we also measured the interaction between cortical delta and cortical beta power from the same region. If the inverse relationship between cortico-basal delta and beta was simply a function of sleep stage depth, we would also expect a strong inverse relationship between cortical delta and cortical beta to be strongly inverted. Unlike correlations between subcortical beta and cortical delta, which were negative for all PD participants, cortical delta and beta showed a weaker negative correlation in only 3 PD participants and a positive correlation in one PD participant (Fig. 3E) as well as in the Dystonia participant during N2/N3 NREM (ON stimulation). LME analysis did show a weaker overall negative fixed effect of cortical beta power on cortical delta power (***β*** = −0.21, 95% CI: [−0.32, −0.1], p-value = 0.0002; n=232,064) during N2/N3 NREM sleep but did not show any fixed group effect of PD/Dystonia state (***β*** = −0.02, 95% CI: [−0.06, 0.004], p-value = 0.1). This indicates that at the cortical level examining the delta/beta interaction, there was no evidence of any difference between the PD/dystonia participants, in contrast to the cortical-subcortical delta/beta interaction. Additionally, in direct model comparison, the LME model for cortical delta with a fixed effect of subcortical beta showed a statistically significant improvement over the model of cortical delta with a fixed effect of cortical beta (simulated likelihood ratio test with 100 replications; p-value = 0.01). This demonstrates that subcortical beta had a stronger effect on cortical delta compared to the relationship between cortical beta activity and cortical delta activity supporting that this subcortical beta - cortical delta effect is greater than any effect of sleep stage depth. Additionally, our data showed that subcortical beta-cortical delta effect was relatively specific to PD.

### Changes in spectral power before spontaneous awakenings

To better understand FP activities at a finer time resolution and investigate the dynamics of intracranial FP that lead to awakenings, we analyzed the change in spectral powers in delta and beta during NREM to wake after sleep transitions. There were a total of 27.4±1.9 awakenings per night from all sleep stages (including N1 to wake transitions) with an average duration of 1.9±0.2 minutes for PD participants during ON stimulation. For N2/N3 NREM specifically, there was a total of 13.5±1.1 N2/N3 NREM to wake after sleep transitions per subject, per night, with each spontaneous awakening averaging 2.7±0.4 minutes for PD participants.

**Figure 4.**
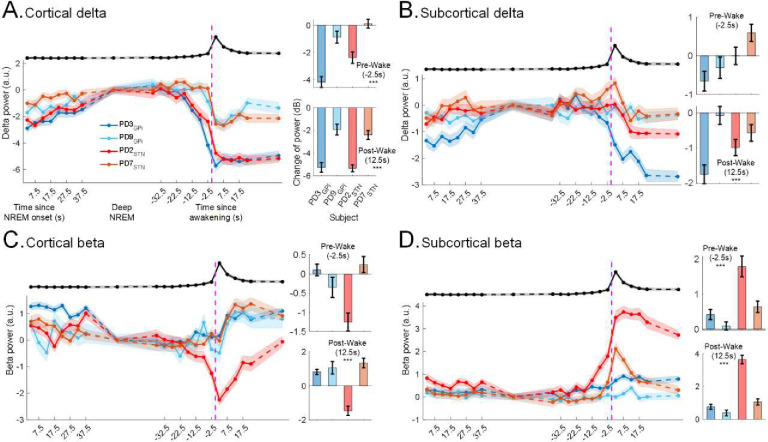
Changes in N2/N3 NREM spectral power before spontaneous awakenings: Subcortical beta increases and cortical delta decreases before spontaneous awakening. **(A)** Cortical delta (1 – 4 Hz) power during N2/N3 NREM to wake after sleep transition episodes for all PD participants (n=4; mean ± SEM) during ON stimulation (*left*). Each data point is the average for 5s data epochs and shadings represent SEMs for N2/N3 NREM to wake after sleep transitions across the recording nights for one participant. Data were pooled from both hemispheres. The vertical purple dashed line shows awakening time. x-axis (*on the left*) shows time in seconds since N2/N3 NREM sleep onset and time since awakening (*middle*, around vertical dashed line). The black line on top shows the across-subject (mean ± SEM) norm of RC+S accelerometry data for all N2/N3 NREM to wake after sleep transitions across all nights for all participants highlighting the awakening time of the episodes. Accelerometry data were rescaled (min-max normalization) in the y-axis (a.u.) for visualization. The bar plots show change in cortical delta power during immediate pre-awakening N2/N3 NREM (2.5s before the wake event, *top*) and early post-awakening (12.5s after the wake event, *bottom*) compared to the average delta power in deep N2/N3 NREM (average over N2/N3 NREM data after 40s from N2/N3 NREM onset and 40s before awakening; SWS). Each bar shows average change of power for one participant and each point shows change of power across all N2/N3 NREM to wake transitions in one night with data pooled from both hemispheres. Cortical delta power gradually increases as sleep deepens and decreases steadily before awakening. The average early post-awakening (12.5s) and immediate pre-awakening N2/N3 NREM delta powers (−2.5s) are lower than those during SWS. The average early post-awakening (12.5s) cortical delta power is lower than the immediate pre-awakening N2/N3 NREM delta power (−2.5s). **(B)** Same as A, for subcortical delta power showing no significant trend across participants or recording sites for both pre and post awakenings. **(C)** Same as A, for cortical beta power showing no significant trend across participants or recording sites for both pre and post awakenings. **(D)** Same as A, only for subcortical beta power illustrating spontaneous rise in N2/N3 NREM beta power before awakenings. The average early post-awakening (12.5s) and immediate pre-awakening N2/N3 NREM subcortical beta power in (−2.5s) are higher than those during SWS.

During N2/N3 NREM, we found that cortical delta power gradually increases as sleep deepens and then subsequently decreases prior to awakening (Fig. 4A) in all participants (multi-night ON stimulation dataset). In our time-resolved analysis, the cortical delta power in the immediate pre-awakening N2/N3 NREM (−2.5s) and early post-awakening (+12.5s) periods were both lower compared to the delta power found in deep NREM stage, (Fig. 4A; immediate pre-wake N2/N3 NREM: ***β*** = −1.8, 95%CI: [−2.3, −1.4], p-value = 7.8e-15; n=886; early post-wake: ***β*** = −3.6, 95%CI: [−4.1, −3.2], p-value = 3.2e-52) and no fixed effects of PD/dystonia condition (immediate pre-wake N2/N3 NREM: p-value = 0.7; early post-wake: p-value = 0.2). This supports that changes in cortical delta during N2/N3 NREM to wake after sleep transitions are not PD specific, but rather a general feature of changes in neurophysiology in NREM sleep versus wakefulness. Conversely, the subcortical delta and cortical beta power also showed statistically significant post wake changes around the time of spontaneous awakenings (early post-wake in subcortical delta: p-value = 1e-10; in cortical delta: p-value = 0.0002; Fig. 4B–C). However, they did not demonstrate pre wake N2/N3 NREM changes that were consistent or significant across participants (immediate pre-wake N2/N3 NREM in subcortical delta: p-value = 0.5; in cortical delta: p-value = 0.08; Fig. 4B–C). However, the subcortical beta power demonstrated a rise before awakenings which was further sustained after awakening (Fig. 4D; immediate pre-wake N2/N3 NREM: ***β*** = 0.6, 95%CI: [0.4, 0.8], p-value = 3.3e-9; early post-wake: ***β*** = 1.2, 95%CI: [1, 1.4], p-value = 1.2e-26) and post-awakening power was higher than pre-awakening N2/N3 NREM (***β*** = 1.2 vs 0.6). Disease state (PD/dystonia) showed a statistically significant fixed effect on the rise of subcortical beta power before awakenings (***β*** = −0.7, 95%CI: [−1.3, −0.2], p-value = 0.006) which was not significant after awakenings (early post-wake: p-value = 0.15) suggesting that the rise of subcortical beta power is relatively PD specific.

### Classifying NREM vs. wakefulness and spontaneous awakenings

We next investigated classification performance using machine-learning (ML) on intracranial neurophysiology to distinguish N2/N3 NREM and wakefulness. To accomplish this, we utilized FP data from the sensorimotor cortical and subcortical regions of 4 PD participants, during the multi-night ON DBS dataset and trained participant-specific support vector machine (SVM) classifiers with six bandpowers features including: delta (0–4 Hz), theta (4–8 Hz), alpha (8–13 Hz), sigma (13–15 Hz), high beta 15–31 Hz) and low gamma (31–50 Hz).

**Figure 5.**
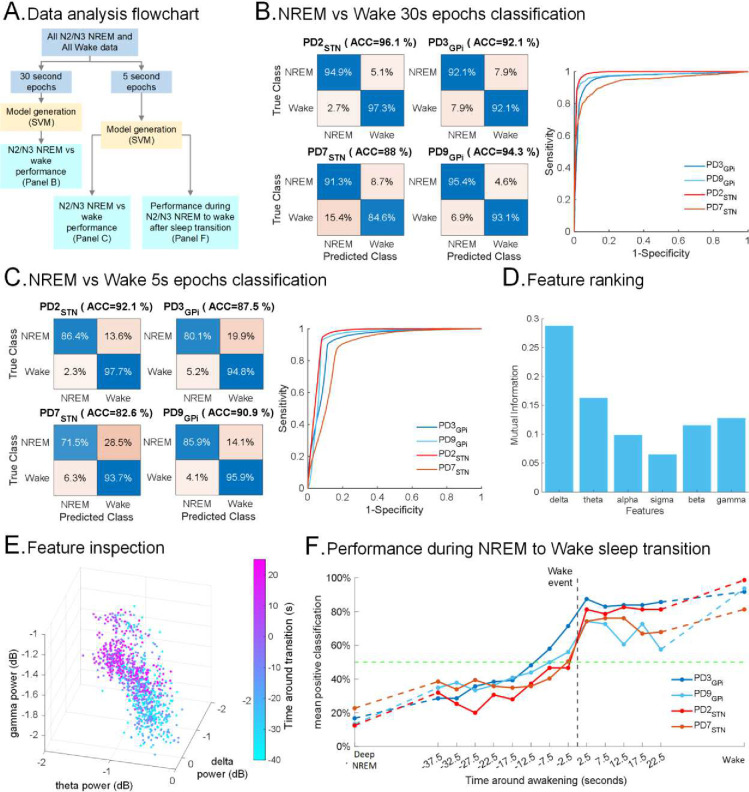
Classification of N2/N3 NREM vs wakefulness with cortical FP: **(A)** Flowchart describing the machine learning (ML) model generation and performance evaluation. **(B)** Performance of participant-specific ML models for N2/N3 NREM vs wakefulness classification for all PD participants (n=4) with classical 30s epoch window in terms of confusion matrices (*left*) and receiver operating characteristic (ROC) performance (*right*). **(C)** Same as B, for 5s epoch window. **(D)** Bandpower feature importance and ranking where x-axis represents 6 bandpower features and y-axis shows average mutual information between bandpower and N2/N3 NREM and wake state across all PD participants (n=4). 5s epochs were utilized. **(E)** Depiction of the top three bandpower features (delta, beta and gamma) in a scatter plot for data from N2/N3 NREM to wake transitions. Data points represent 5-second epochs from a single PD participant (PD2). Color bar (*left*) shows the time around awakening in seconds. **(F)** Performance of the ML models trained on 5s epochs shown in C during N2/N3 NREM to wake after sleep transition. The x-axis represents time in seconds around awakening and y-axis is the average wake classification by the ML models across all transitions of the participant. The vertical black dashed line shows awakening time and the horizontal green dashed line represents 50% average wake detection by the models. For all panels, left and right side data were pooled. For ground truth of 5s epochs, actual awakening events within the classical 30s sleep epochs were determined with accelerometry data and then segmented into N2/N3 NREM and Wake 5s segments (see Methods and Supplementary Fig. 3).

First, we trained a participant-specific SVM model for each PD participant (n=4) in classical 30s time windows to classify all N2/N3 NREM vs wake epochs using first cortical and then subcortical features (Fig. 5A). Our results showed strong classification performance both using sensorimotor cortex and subcortical regions (Cortex: 92.6±1.7% accuracy, 91.8±2.6% sensitivity, 93.4±1% specificity and 96.6±1% area under the curve (AUC); STN/GPi: 86.7±3.5% accuracy, 84.8±4.2% sensitivity, 88.6±2.9% specificity and 93.1±2.3% AUC; Fig. 5B; Supplementary Fig. 5A; Supplementary Table 2; Supplementary Table 3) for N2/N3 NREM vs wake classification across all PD participants at the standard 30s epoch level. We then probed N2/N3 NREM vs wake classification at a finer temporal scale (reducing from 30s to 5s epoch) for rapidly dynamic awakening detection-towards sleep-specific aDBS development. Specifically, we trained participant-specific SVM models using 5s epochs achieving again high classification performances (Cortex: 88.3±2.1% accuracy, 95.5±0.8% sensitivity, 81±3.5% specificity and 92.5±1.5% AUC; STN/GPi: 80.1±4.5% accuracy, 88.7±2% sensitivity, 71.6±8.4% specificity and 88±3.7% AUC; Fig. 5C; Supplementary Fig. 5B; Supplementary Table 2; Supplementary Table 3) for N2/N3 NREM vs wakefulness. This highlights the potential for rapid sleep staging using intracranial neurophysiology and faster time scales than traditional 30s epochs. Feature ranking using mutual information revealed that the top cortical feature for N2/N3 NREM versus wakefulness distinction was delta power while the top subcortical feature was beta power (Fig. 5D; Supplementary Fig. 5C), aligning with our earlier findings of cortical delta - subcortical beta interactions in spontaneous awakenings. Three top-ranked features for epochs near the awakening events revealed a gradual distribution of features in the manifold between N2/N3 NREM and wakefulness during the N2/N3 NREM to wake after sleep transitions (Fig. 5E; Supplementary Fig. 5D) supporting a gradual shift of the classifier output from N2/N3 NREM to awakening. Finally, we assessed the time-resolved performance of the ML classifier during transitions from N2/N3 NREM to wake after sleep transitions. The average wakefulness classification of the ML models during the N2/N3 NREM to wake after transitions showed a gradual increase in wake detection during the hypnogram and accelerometer defined awakening events (Fig. 5F). Specifically, we found low wake detection during sustained N2/N3 NREM (sustained N2/N3 NREM: 16.3±2.3%) and more than 50% wake detection using cortical bandpower features even preceding (t=−2.5s; 56.1±5.4%) the awakening events despite the low temporal resolution of the classical hypnogram (updated at a 30s sampling rate). Classification further improved shortly after the hypnogram and accelerometry defined wakening events (t=+2.5s; 79.32±3.2%; sustained wake: 91.34±3.6%) across all PD participants.

## Discussion

We collected multi-night intracranial cortico-basal neural recordings from five participants (four PD and one dystonia) from cortical and subcortical regions, paired with polysomnography for both DBS ON and OFF conditions, remotely in participants’ own homes over 58 nights. We found increased cortio-basal slow wave and decreased beta activity as well as changes in cortico-basal functional connectivity during N2/N3 NREM, an effect that was enhanced by DBS. Within N2/N3 NREM, there was a direct inverse relationship between subcortical beta and cortical delta activity and further, we found that subcortical beta power rose prior to spontaneous awakenings at high temporal resolution (5s). These data support the hypothesis that subcortical beta is related to overnight sleep disruptions and spontaneous awakenings in PD. Finally, we utilized ML models on cortico-basal intracranial data and achieved high performance in classifying N2/N3 NREM and wakefulness both in the classical (30s) and in sub-classical rapid (5s) time windows, providing a foundation for future personalized sleep adaptive DBS.

Our study advances understanding of sleep neurophysiology in a number of areas. First, technically, we recorded full spectrum, time-domain, intracranial cortical and subcortical neural activity during sleep, over multiple nights (n=58), using chronic electrodes and a fully embedded, sensing-enabled, DBS device in the naturalistic setting from participants in their own homes. Second, we provided evidence of subcortical beta and cortical delta interaction during NREM in PD participants and its modulation by DBS, within participants at high temporal resolution (5s). This effect has been previously noted in a primate model of PD^17^, but to our knowledge, our study is the first to demonstrate this interaction within humans within subjects with PD. Third, this was supported by analyses of NREM sleep neurophysiology in both ON and OFF stimulation which revealed both stimulation-dependent (increased cortical delta and decreased alpha plus low beta in N2/N3 NREM) and stimulation-independent (subcortical beta and cortical delta interaction) effects. Our cortico-basal delta beta interaction findings and pre-awakening subcortical beta rise had significantly stronger effects in the PD cohort in our comparison with our dystonia participant. Finally, we build tractable, constrained, ML models that can distinguish spontaneous awakenings on a rapid timescale.

It is established that during the daytime, subcortical beta oscillations are excessive in PD and potentially contribute to circuit disruption and motor symptoms^29,30^. Here, we show that subcortical beta oscillations also disrupt cortical slow oscillations during N2/N3 NREM sleep in humans with PD and are partially responsible for awakenings during the night, validating findings from PD models in primates^17^. Further, we show that DBS stimulation, while known to reduce subcortical beta oscillations during wakefulness^31^, also significantly impacts sleep neurophysiology resulting in the increase in cortical delta power and a decrease in cortical alpha and low beta during N2/N3 NREM sleep. This finding aligns with previous studies where an increased accumulation of EEG delta power during NREM sleep was found as a result of subthalamic DBS in PD^32^, but here provides a candidate causal mechanism. Data in our study indicates that DBS therapy appears to improve sleep in PD, at least in part, through direct modulation of beta and delta oscillations.

Although previous studies have documented the presence of subcortical beta oscillations during sleep in STN and GPi^18–22^, these studies have been single-night studies, recorded in externalized patients post-operatively in a laboratory. Further, previous studies have not been able to probe interactions between intracranial subcortical beta and cortical delta, spontaneous awakenings during the night nor investigated DBS stimulation effects on sleep physiology with the level of temporal precision presented here. In this study, we show that within NREM sleep, subcortical beta inversely correlates with cortical delta power and precedes spontaneous awakenings on a fast time scale. Our findings on the mechanisms of cortical-subcortical interactions during sleep provide a foundation for the development of adaptive DBS approaches for restoring normal sleep patterns in people with PD.

The link between sleep dysfunction and daytime motor, mood and cognitive symptoms makes sleep an enticing potential target for further investigation^5–7^. Moreover, sleep disturbances, and particularly reductions in cortical slow wave activity during NREM have been linked to faster disease progression^11,14^. Therefore, restoring NREM sleep architecture with adaptive DBS has the potential to reduce overnight insomnia, improve waking motor and non-motor symptoms and increase cortical slow waves that could impact disease progression. This supports the proposal that daytime neural activities and overnight sleep physiology are notably dissociable and require different strategies for aDBS to optimize rhythms during these two distinct phases. Implementing different aDBS algorithms around the circadian cycle could be achieved by the introduction of daytime (versus sleep) neural classifiers, circadian (clock) based algorithms and combined feedforward and feedback controllers that optimize both daytime and nighttime neurophysiology^33,34^.

Our ML analyses demonstrate N2/N3 NREM vs wakefulness classification not only in classical 30s sleep epochs but also at rapid time windows (5s) with high accuracy. The ML models showed increasing wake detection around the actual awakening events using intracranial brain recordings (Fig. 5F). In light of the low temporal resolution of the precise timing of awakening events (classified using the 30s hypnogram epochs), this gradual increase likely significantly reflects jitter in the precise awakening time and therefore represents a floor on potential classification accuracy. The performance of these ML models, despite being constrained to simpler ML algorithms (with potential for embedding on emerging DBS devices) and having only limited power band feature inputs, suggests the viability and potential applications of machine-learning algorithms for identifying micro-stages of sleep and designing adaptive DBS therapies that can modulate stimulation to prevent awakening.

Limitations include the fact that our ground-truth sleep stage labelings were obtained through a portable polysomnogram and automated sleep-scoring algorithm, validated on healthy controls^26^, instead of a conventional laboratory-based PSG. However, we note that our intracranial recordings, grouped according to sleep stages defined from our portable PSG, revealed anticipated and classical changes in cortical (ECoG) activities across various stages (Fig. 1F–G, Supplementary Fig. 4). In particular, the observed elevation in cortical delta power during N3 sleep and reductions in beta power provide evidence of the differentiation of underlying sleep stages within our group of participants using this pipeline (Fig. 1F–G, Supplementary Fig. 4). Furthermore, our portable remote setup enabled us to collect multi-night recordings in a natural setting which compares favorably to single-night PSG recordings (from a sleep laboratory) which can be subject to first night acclimatization and sleep disruption effects. We also report many nights of recordings per participant (n=58 total), but from a relatively small number of subjects, which supported highly statistically powered LME analyzes that modeled within, as well as across, participant effects - similar to the strengths of primate research. This approach was felt to be well suited to looking for the within-subject cortico-subcortical interactions which were the primary focus of this study. However, evaluation of across-subject factors, including analysis of how beta-delta interactions predict clinical outcomes at scale would require a different study design that included large numbers of subjects (but would also require much less within-subject data). Finally, our comparison participant was a single cervical dystonia patient (rather than a formal control group) reflecting the uniqueness of this participant cohort, with high-resolution sensing-enabled pulse generators and chronically implanted ECoG electrodes. However, despite this, and in view of the large within-participant dataset size and linear mixed modeling, we were able to show a difference between the dystonia participant and the PD group, which should be supported in the future by larger and more balanced cohorts. In this study, we restrict our analysis to NREM and canonical power bands with a focus on beta and delta^28^. We did not examine changes in other sleep stages or specifically analyze sleep spindles (which overlap in frequency with low beta) or other frequency bands which will be reported separately. Finally, our ML models in this study were limited to straightforward binary classification (N2/N3 NREM vs. wakefulness) with a view towards the constraints of current and emerging sensing-enabled DBS devices.

## Conclusions

In this study, we recorded and analyzed intracranial FPs with extracranial polysomnography at-home over multiple nights in PD participants, in the presence and absence of DBS. Our data revealed that cortico-basal network spectral power and connectivity in the delta and beta bands are increased and decreased in N2/N3 NREM versus wakefulness respectively, an effect that was enhanced by DBS. Further, within N2/N3 NREM, cortical delta band slow wave activity was inversely related to subcortical beta, which also rises prior to spontaneous awakenings. Finally, our machine-learning models achieved high accuracy in distinguishing between N2/N3 NREM and wakefulness, both in classical and a faster time scale. These findings uncover a role of subcortical beta in sleep dysfunction in PD and provide targets as well as machine-learning models for future personalized sleep-specific adaptive DBS.

## Methods

### Participants, demography, and ethics

We recruited 4 participants with idiopathic PD for this study (Table 1). A movement disorders physician diagnosed each individual with PD according to the Movement Disorder Society PD diagnostic criteria^35^. The motor component of the United Parkinson’s Disease Rating Scale (UPDRS) scores were administered by trained raters. We also recruited one participant with cervical dystonia as a comparison participant. Participants were recruited from a parent study focused on investigating closed-loop DBS for daytime motor symptoms. Implanted electrodes were connected to an investigational sensing-enabled Summit RC+S DBS implantable pulse generator provided by Medtronic (Fig. 1A)^24^. This study was reviewed by our Institutional Review Board and registered on clinicaltrials.gov (NCT0358289; IDE G180097). The study was also reviewed by the Human Resources Protection Office (HRPO) at Defense Advanced Research Projects Agency (DARPA). Written informed consent was provided by all participants. All participants had chronic bilateral cortical ECoG electrodes and two PD participants were implanted with bilateral electrodes in the Subthalamic Nucleus (STN; PD2 and PD7) and two PD participants along with one dystonia participant were implanted with bilateral electrodes in the Globus Pallidus (GPi; PD3, PD9 and dystonia participant) nuclei (Fig. 1B). DBS electrode implantation targets were determined by the clinical team. A movement disorder specialist programmed the participants with conventional DBS settings, optimizing stimulation to address daytime motor symptoms.

### Experimental design and protocols

We collected data using two protocols: long-term multi-night data collection ON stimulation plus separate two night comparison recordings, one night ON DBS and one night OFF DBS. During the long-term overnight data collection, each participant (n=5) was equipped with a portable PSG (Dreem2) headset and overnight intracranial data as well as polysomnography data were recorded for ~10 nights (Supplementary Table 1) that were predominantly consecutive. In the ON/OFF protocol, overnight data from the PD participants (n=4) were collected for two consecutive days. On the first day DBS was ON (3.075 ± 0.65 mA) and the next day DBS was OFF. During both data collection protocols, the PD participants were on their regular clinical dopaminergic replacement medications. ON/OFF recordings were not completed in the cervical dystonia participant at the participant’s request. All data recordings were performed remotely in participants’ homes.

### Polysomnography acquisition

Extracranial polysomnography (PSG) was recorded through the Dreem headband which includes an automated sleep staging algorithm with extracranial electroencephalography (EEG) data (Dreem2 headband, Dreem Co., Paris, France)^26,27^. The Dreem2 headband provided sleep stage classification hypnograms according to scoring methods (NREM: N3, N2, N1 and REM) of the American Academy of Sleep Medicine (AASM) which has been validated on healthy participants (Fig. 1C)^26,27^. The sleep staging was performed using EEG data at every 30s epoch. Sleep onset was defined as the start of the NREM sleep (3 consecutive epochs were required to classify N1). Wakefulness after sleep onset (WASO) was calculated as the total waking time after sleep onset and before the last epoch of sleep. As N1 is difficult to detect and physiologically distinct, we focused our analysis on N2 and N3 stages for NREM sleep (denoted as N2/N3 NREM).

### Intracranial data collection

For each participant, the Summit RC+S device was implanted bilaterally and connected to bilateral sensing and stimulation-capable quadripolar leads in the basal ganglia targets (STN in 2 PD participants or GPi in 2 PD participants and 1 cervical dystonia participant) plus quadripolar sensorimotor chronic electrocorticography (ECoG), sensing only strips, with 4 electrode contacts spanning the central gyrus (Fig. 1B). Overnight intracranial data were collected from cortical and subcortical structures in both left and right hemispheres (Fig. 1D) in addition to data from bilateral accelerometers embedded within the chest-mounted pulse generator devices. The time series FP data were recorded at either a 250 Hz or 500 Hz sampling rate.

### Data Preprocessing

The intracranial recordings were validated and synchronized to the PSG recordings using accelerometry data. Cross-correlation was applied to accelerometry data from both the Dreem2 band and the RC+S neurostimulator in order to ascertain the delay between PSG and RC+S time series (Supplementary Fig. 3). As PSG hypnogram sleep stage estimates were performed on 30s epochs, we also used post awakening movement (measured via accelerometry) to further re-align hypnograms to awakening events at sub-classical 5s epochs (Supplementary Fig. 3). All intracranial data were downsampled to 250 Hz and filtered through a 0.8–100 Hz zero-phase IIR elliptic bandpass filter with 1dB passband ripple and 100 dB attenuation (‘filtfilt’ and ‘designfilt’ function in Matlab; Fig. 1E). Large artifactual spikes in the subcortical intracranial data were removed along with the corresponding cortical data (Supplementary Fig.3). To identify artifacts, absolute squared subcortical data were first smoothed with a Gaussian kernel with 1s window then any period larger than 5 times the median over the whole night was considered artifactual spikes. The ECG artifacts in the subcortical data were removed using a combination of two ECG data remover algorithms (‘PerceptHammer’ and ‘Perceive’ library; Matlab; Supplementary Fig. 3)^36,37^.

### Power spectrum analysis

To calculate the power spectra, the intracranial data from each night were z-scored for each location. Then, the N2/N3 NREM data segments were collected together according to the PSG hypnogram labels. The selected data were segmented into 5s epochs and power spectra were calculated for each epoch using a Hamming window of 1-s, 512 point FFT with 50% overlap by Welch’s method (‘pwelch’ in Matlab) which was normalized by the total power in 0–50 Hz. The calculated power spectrums for each epoch were then pooled over both hemispheres within participants. For calculating the change in power spectrum in N2/N3 NREM with wakefulness as the baseline, the power spectrums for wake epochs were calculated in a similar manner as during N2/N3 NREM and the difference between the average wake power spectrum and N2/N3 NREM power spectrum for each night was calculated. For calculating the ON vs OFF power spectrum, average power spectra were calculated for ON and OFF nights and their difference was taken. The averages were calculated on log-transformed power spectra.

### Spectral coherence analysis

To compute the spectral coherence, the intracranial data obtained from each night were normalized using z-scoring for each location. Subsequently, N2/N3 NREM data segments were extracted and then divided into 5s epochs. For each epoch, 5s of cortical and subcortical data were utilized for estimating the one-sided magnitude squared coherence using the multitaper method (‘mscohere’; Matlab) with Hamming window of 1-s and 512 point FFT. The epoch-wise spectral coherences were then pooled over both hemispheres. Similarly to the power spectral analysis, spectral coherences for wake epochs were calculated and the difference between the average of wake and N2/N3 NREM spectral coherence for each night was calculated in order to obtain the change in spectral coherence in N2/N3 NREM with wake as the baseline.

### Beta-delta correlation analysis

To analyze the interaction between subcortical beta and cortical delta activity during N2/N3 NREM sleep in intracranial signals, we applied z-scoring, power spectrum calculation and normalization techniques as previously described. However, there was one exception regarding the normalization of the cortical power spectrum where instead of normalizing it by dividing the total power (0–50 Hz), we divided it by the total power excluding the beta range (0–13 and 31–50 Hz). This adjustment was necessary to avoid detecting spurious negative correlations that could be caused by the normalization procedure itself. Both subcortical beta and cortical delta were calculated for 5s epochs which were log-transformed for each night and each hemisphere. The band powers were then pooled over both hemispheres. Subsequently, for each participant, we calculated the Spearman’s rho correlation coefficient between subcortical beta and cortical delta power across all 5s epochs for each night. For calculating the delay between subcortical beta and cortical delta power, normalized cross-correlation (‘xcorr’ function in Matlab) was calculated between these band powers from the 5s epochs from above for each night. Lag was calculated by finding the minimum (trough, reflecting a negative relationship) normalized cross-correlation between the two band powers. Epoch band powers for each night were smoothed using a 20-point Gaussian kernel. Data for each night were mean-subtracted and pooled from both hemispheres. To investigate interactions between delta and beta powers from cortex, we applied the same power spectrum calculation techniques on 5s epochs as previously described in beta-delta correlation analyses. The only exception was the normalization step of the power spectrum which was not applied to avoid detecting artificial negative correlations that could be imposed by the normalization of the power spectrum. Spearman’s rho correlation coefficient was calculated between the cortical delta and beta power in all 5s epochs throughout each night for all participants.

### NREM to wake after sleep transition analysis

To investigate the changes in spectral power during N2/N3 NREM to wake after sleep events, all intracranial data were bandpassed using the zero-phase IIR elliptic bandpass filters. Next, the data were z-scored for each night for each hemisphere at all locations. Hilbert transform was applied to the z-scored data and the absolute squares of the results were converted into a decibel scale for band-specific power. After detecting all N2/N3 NREM to wake after sleep transitions, events with N2/N3 NREM sleep less than 85s and wake periods of less than 25s were ignored. Band-power for each 5s epoch was averaged. The time label for each 5s epoch was found by taking the average of the start time and stop time of the epoch (e.g., epoch from 0s to 5s was labelled as 2.5s). All epochs in N2/N3 NREM data after 40s from NREM onset and 40s before awakening were averaged to calculate deep NREM (slow-wave sleep; SWS) power. All epochs in awake stage data after 25s from wake onset were averaged to calculate awake stage power. All data were analyzed from the ON stimulation multi-night dataset.

### Machine-learning models

For both 30s and 5s epoch windows, individual SVM models (‘fitcsvm’ in Matlab) with Gaussian kernel were trained for each PD participant. For class balance, we randomly reduced the number of N2/N3 NREM epochs (using ‘datasample’ in Matlab) to match the total number of wake epochs. To calculate the bandpower features, we first standardized the intracranial data for each location across each night using z-score. Then, the N2/N3 NREM and wake stage data were collected based on the PSG hypnogram labels. These data were segmented into 30s and 5s epochs and power spectra were calculated for each epoch using Welch’s method with a 1s Hamming window and a 512-point FFT, along with a 50% overlap (‘pwelch’ in Matlab). Uniform prior distribution was assumed during training and no score transform was applied. Kernel width was 2.6 and the standardization of the features were enabled. 5-fold cross-validations were performed for observing the N2/N3 NREM vs wake classification performances. For the models using 5s epochs, customized cost function was applied during the training to make the models more sensitive to wake events. The threshold for binary classification was 0.5 and no further threshold optimization was conducted. For feature importance and ranking, the mutual information between bandpower features of 5s epochs and N2/N3 NREM vs wake class labels were calculated^38^. Similar feature ranking results were obtained using 30s epochs. Data from left and right hemispheres were pooled for all analyses.

### Statistical methods

A significance threshold of 0.05 was employed to determine statistical significance. Linear mixed effect models (LME) were utilized (‘fitlme’ in Matlab) for investigating the spectral power and coherence differences, the interactions between cortical and subcortical beta with cortical delta powers. For LME models, full covariance matrix was applied using the Cholesky parameterization which estimated all elements of the covariance matrix. We accounted for the correlation between 5s epochs within the same night by including ‘night #’ as a random effects term in our LME models. Theoretical likelihood ratio test (‘compare’ in Matlab) was used for comparing LME models. Wilcoxon rank sum test (‘ranksum’ in Matlab) was utilized for measuring group-level differences in wake predictions. All analyses were performed using Matlab 2022a (Mathworks).

## Figures and Tables

**Table 1:** Participant demographics

Participant ID	PD2	PD3	PD7	PD9	Dystonia

Age	58	66	40	48	65
Gender	M	M	M	M	M
Diagnosis	PD	PD	PD	PD	Dystonia
Dx	11	13	9	13	30
Stim Target	STN	GPi	STN	GPi	GPi
Pulse Width (us)	60	60	60/90	90	60
Stim amp. (mA)	L: 2.4 R: 3.1	L: 3.7 R: 2.8	L: 1.7–3.4 R: 1.7–3.4	L: 3–3.7 R: 3–3.7	L: 4.5 R: 3.5
Stim freq. (Hz)	130.2	178.6	130.2	150.6	130.2
Stim contact	L: C+2- R: C+1-	L: C+1- R: C+1-	L: C+2- R: C+2-	L: C+2- R: C+2-	L: C+1- R: C+2-
Medication details	A-HCL 100mg (3 times daily) C-Ldopa 25–100 mg IR (5 times daily)	C-Ldopa 25–100mg CR (1–2 tabs at bedtime) and 25100mg IR (3 times daily)	C-Ldopa 25100mg (1 time daily) Rasagaline (Azilect) 1mg (1 time daily)	Rytary 195mg (3 times daily)	-
UPDRS-III (OFF)	49	66	41	39	-
UPDRS-III (ON)	5	24	14	16	-
UPDRS 1.7	No sleep symptoms	Slight sleep symptoms	Slight sleep symptoms	Mild sleep symptoms	-
UPDRS 1.8	No daytime sleepiness	Mild daytime sleepiness	Moderate daytime sleepiness	Mild daytime sleepiness	-
Sleep diagnosis	No sleep conditions	Nocturia, RBD	Daytime sleepiness	OSA, Insomnia	Restless Leg Syndrome
Neuropsych report (pre-op)	No reported sleep disorder or conditions	Mild sleep difficulties, with nocturia and RBD	Day time sleepiness (strongest 4–5pm), usually sleeps late and sleeps very little overnight	Had long term difficulties sleeping before PD. Occasionally couldn’t fall asleep at night.	Good sleep. No movements/dystonia at night. Restless Leg Syndrome at night.

UPDRS scores were pre-op, Dx= Disease duration (Years), RBD= REM sleep behaviour disorder, Obstructive sleep apnea = OSA, PD = Parkinson’s disease, R = Right, L = Left, C-Ldopa = Carbidopa-Levodopa (Sinemet), A-HCL = Amantadine HCL (Symmetrel), Stim = DBS stimulation. None of them suffered from Dementia.

## Data Availability

Datasets and codes generated and/or analyzed in this study will be shared upon reasonable request for reviewing purposes only. These datasets are part of an ongoing medical research project.
